# The Impact of Changed Strategies for Patients with Cholangiocarcinoma in This Millenium

**DOI:** 10.1155/2015/736049

**Published:** 2015-02-18

**Authors:** Per Lindnér, Magnus Rizell, Lo Hafström

**Affiliations:** Transplant Institute, Institute of Clinical Sciences, Sahlgrenska Academy at University of Gothenburg, Sahlgrenska University Hospital, SE-413 45 Gothenburg, Sweden

## Abstract

*Background.* Cholangiocarcinoma is a cancer with a poor prognosis. In this millennium there are new diagnostic and therapeutic strategies for these patients. *Aim.* The aim of this study was to find if these changes influenced survival of individuals with proximal cholangiocarcinoma. *Material.* 627 individuals with a diagnosis of cholangiocarcinoma (not including distal common duct cancer) during the period from 2000 to 2011 were registered in Sweden's Western Region. The material was divided into three consecutive time periods. *Results.* The overall survival curves for individuals with cholangiocarcinoma improved over the three time periods (*n* = 627) (*P* = 0.0013). Median survival increased from 2.6 months in the first period (2000–2003) to 3.6 months in the final four years (2008–2011). Patients with perihilar cholangiocarcinoma (PHC) had longer median survival than those with intrahepatic cholangiocarcinoma (IHC): 6.8 versus 3.2 months (*P* = 0.0003). An improvement in the survival curves over time was seen for those with IHC (*P* = 0.034) but not for patients with PHC (*P* = 0.38). Nine percent of the patients with IHC had potential curative surgical therapy. The three-year survival rate after liver resection for patients with IHC was 35% and 60% after liver transplantation. Among patients with PHC, 15.3% had potential curative bile duct resection with a concomitant liver resection and 6.1% bile duct resection alone. The three-year survival rate for these two groups was 32% and 20%, respectively. *Conclusion.* Overall survival for individuals with PHC was better than for those with IHC. Over time survival in IHC patients improved but not in those with PHC.

## 1. Introduction

Analysis of a complete population of individuals with a defined cancer diagnosis over a prolonged time period is needed to assess whether new therapeutic strategies have affected the whole population's survival outcome.

Cancers arising from the epithelial lining of the intrahepatic, perihilar, and extrahepatic bile ducts are a heterogeneous group of malignancies. Two major diagnoses are identified by location: the intrahepatic cholangiocellular cancer (IHC) and the perihilar carcinoma (PHC) or Klatskin tumour at the confluence or bifurcation of the left and right hepatic duct proximal to the cystic duct. There is not a clear-cut border between an advanced PHC and an IHC.

The prognosis for individuals with these conditions is poor. An analysis of Netherlands Cancer Registry record for IHC found a three-year survival rate of 8%, but a steady increase over the past decades [1]. This change could be the result of developments in surgery, transplantation, and the introduction of new ablative and molecular targeted therapies.

Bile duct resection with or without liver resection is the hallmark of potentially curative treatment for patients with PHC and 5-year survival is reported among 20–40% after surgical resection [2].

Most of these reports describe highly selective materials subjected to surgery in referral centres and it is difficult to assess if these results affect the whole population with the disease [3–5].

For patients with IHC liver resection can achieve up to 40% five-year survival [6]. With stringent inclusion criteria liver transplantation according to the Mayo protocol has shown impressing long-term results, in PHC, even in multi-institutional setting [6, 7].

The combination of gemcitabine and cisplatinum [8] has demonstrated a survival advantage over treatment with gemcitabine alone, suggesting that certain chemotherapies add to survival [8].

Survival for a complete population of patients with a malignant disease is improving thanks to advancements in diagnostic procedures, patient managements, surgical procedures, and emergence of effective chemotherapeutic agents and molecular target drugs. All of these factors are relevant for the improvements of patients' outcome.

The hypothesis of this study was that there is a continuous improvement in survival for the total population of patients with cholangiocellular carcinoma in this millennium.

The study's goal was further to analyse whether staging and/or therapy options used during the first decade of this century have had an impact on the outcome. The population studied consisted of all individuals diagnosed as having IHC or PHC served by the Western Regional Cancer Centre (RCC) in Sweden. The hypothesis that there is an improvement in survival for patients with cholangiocarcinoma was formulated before data collection.

## 2. Material

The region served by the RCC has a population of 1.6 million. In this region there is one referral university hospital having a complete liver cancer service, including liver transplantation. All individuals with an established histopathological cancer diagnosis are reported to the RCC. All patients in whom a diagnosis is clinically established are also reported to the register. Included in report data is the stage of the cancer based on clinical and imaging findings, which are translated into TNM criteria (TNM 6 or higher).

In this study's analysis, clinical and pathological information was retrieved from RCC records. Additional information was obtained by reviewing individual patient's charts. When several therapeutic procedures were described the most important therapy was ranked as the instituted therapy. Data on pre- and postoperative adjuvant therapy was not collected. Active palliative treatment (APT) included chemotherapy, TACE (transarterial chemotherapy), sirolimus, sorafenib, and COX-2 inhibitors. Bile duct drainage and radiation therapy of skeleton metastases were considered as best supportive care (BSC).

All curative and active palliative treatments were handled by the liver surgery service at the university hospital.

Cholangiocarcinomas located distally in the bile duct were not included in the analysis.

Between 2000 and 2011 a total of 627 individuals were reported to the RCC as having cancers originating in the epithelial lining of the bile ducts. The 49 individuals, where a diagnosis was not established until the postmortem examination, were also excluded. Available information about the clinical staging, treatment, and survival on the remaining 578 individuals was reviewed. Morphologic diagnosis of cholangiocarcinoma was established when an image of adenocarcinoma was present in the specimen, in combination with immunochemical markers typical for cholangiocarcinoma, including CD7 and CD17. Differentiation between IHC and PHC was based on the location of the tumour within the liver.

A number of patients were at an advanced stage when diagnosed with the cancer and in these patients only minor diagnostic procedures were motivated. These patients were registered under the diagnosis of unspecified primary liver cancer (*n* = 65) or unspecified bile duct cancer (*n* = 117) (Table 1). The diagnosis of unspecified primary liver cancer supposedly cholangiocellular (mainly ICD-10: C22.9) used by the register was based on the following: (1) no evidence of a previous or concomitant cancer of no hepatic origin, (2) no underlying liver disease, and (3) no signs of hepatocellular cancer were found. The diagnosis of unspecified bile duct cancer (mainly ICD-10: C24.9) was based on the dominant symptom of stricture(s) in the extra hepatic bile tree above the gallbladder duct that was causing jaundice. These two diagnoses inevitably included mainly cases of IHC and PHC where only best supported care (BSC) was administered and median survival was less than two months. These groups are not further analysed.

In order to explore if there was a continuous progress in the outcome, the material was divided into three equal time cohorts: Period A 2000–2003, Period B 2004–2007, and Period C 2008–2011. No planned changes in the organization of hepatobiliary surgery occurred in the region during 2000–2012, but there was an increased awareness of the disease, which led to more referrals (Table 1).

### 2.1. Statistics

Survival time was calculated from the date of the report to the RCC, that is, the date a diagnosis was established histopathologically or clinically. Observation time was more than 32 months or till death in all cases. Survival estimates were made using the Kaplan-Meier method and compared using the log-rank test. All statistics were calculated using SPSS 22 Statistical Software (SPSS, Chicago, IL) and at a significance level of 5%.

### 2.2. Ethics

The analyses were done at Transplant Institute, Section for Liver Surgery, Sahlgrenska University Hospital, Gothenburg, Sweden, and Regional Cancer Centre of West Sweden, Gothenburg, Sweden. The study was carried out in compliance with the Declaration of Helsinki principles.

## 3. Results

**Figure 1:**
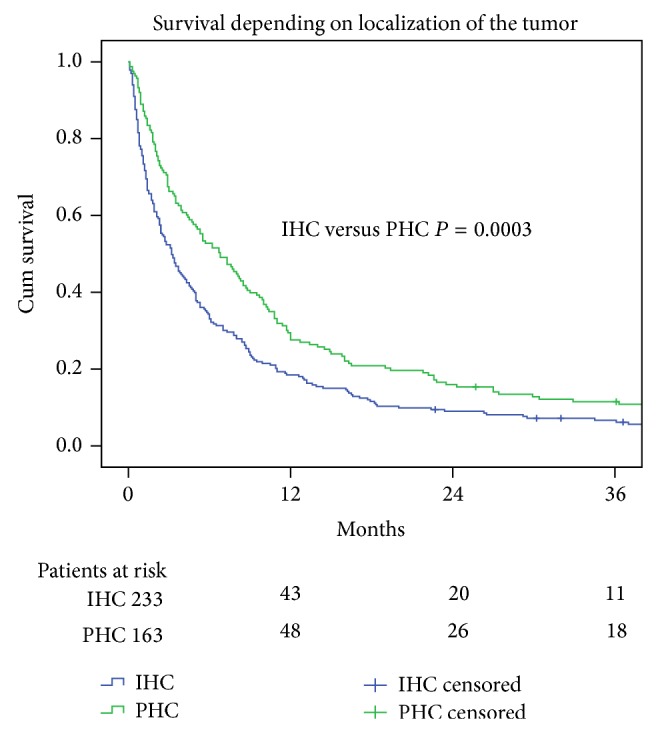
Survival curves (Kaplan-Meier log-rank test *P* = 0.0003) for 396 patients with cholangiocarcinoma. Perihilar cancer (PHC) (C 24.0); intrahepatic cancer (C 22.1). Median survival (95% confidence interval) in months: PHC 6.80 (4.81–8.78) and IHC 3.20 (2.30–3.10).

Overall survival for all individuals with cholangiocarcinoma (*n* = 578) improved over time (*P* = 0.0013). The survival curves for the IHC and PHC are shown in Figure 1.

The median survival for patients with PHC was 6.8 months and for those with IHC 3.0 months (*P* = 0.0003).

The age and sex distribution did not change between the three time periods for neither IHC nor PHC.

### 3.1. Intrahepatic Cholangiocellular Cancer (IHC)

74% of the individuals with IHC were between 50 and 79 years of age when diagnosed, with an equal number younger (13%) and older (13%) than these age groups. The clinical staging for the 233 individuals with IHC in the three time periods is depicted in Table 1(a). One patient was staged as T0 and was transplanted for primary sclerosing cholangitis and IHC was identified in the explanted liver. Over time an increasing number of patients were clinically staged (63% in Period A versus 94% in Period C). Among the staged patients with IHC there was no staging migration over time (*P* = 0.27). In 17% an underlying liver disease was identified. Of those 40 patients ten had primary sclerosing cholangitis (PSC). The diagnosis IHC was established morphologically in 91% of the patients. The diagnosis was in 27% supported by immunochemistry, CD 7 and CD 19.

**Figure 2:**
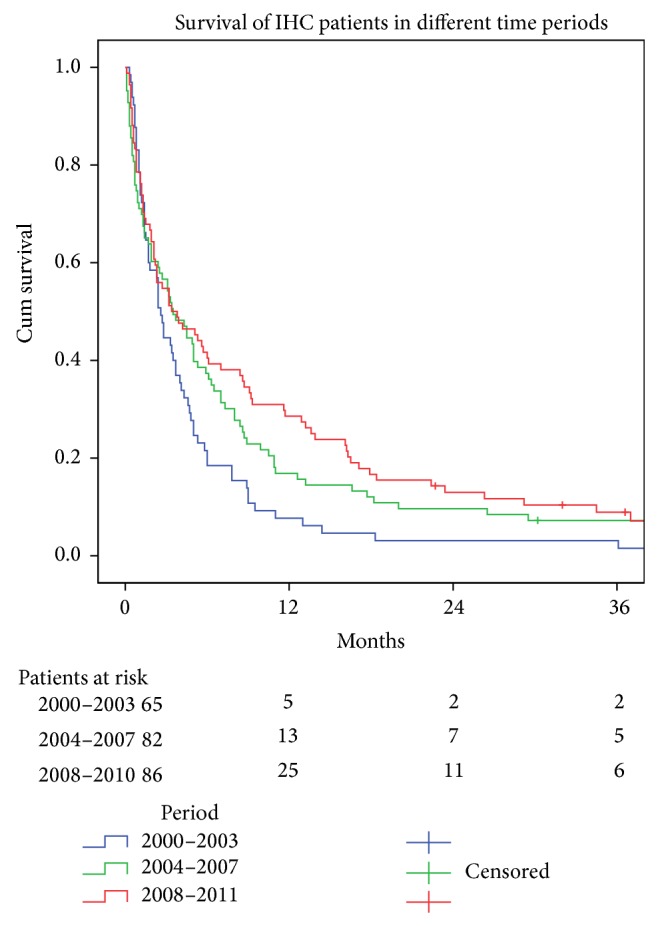
Survival curves (Kaplan-Meier log-rank test *P* > 0.03) for 233 patients with intrahepatic cancer (IHC) for three different time periods. Median survival (95% confidence interval) in months: 2000–2003, 2.60 (2.11–3.09); 2004–2007, 3.50 (1.89–5.11); and 2008–2011, 3.40 (0.71–4.07).

Overall survival for individuals with IHC was 3.2 months and 6.6% survived more than three years. A significant improvement in overall survival over time was registered for those diagnosed as having IHC (*P* = 0.034) (Figure 2). The one-year survival was significantly higher in the last time period compared to the first period (*P* = 0.038). After two years this difference was no longer significant (*P* = 0.34).

Seventeen of 233 patients (7.3%) had a liver resection and five had a liver transplantation. The three-year overall survival after liver resection was 29% and after liver transplantation 60%. There was a nonsignificant increase in the number of liver resections and transplantations between Periods A and C (*P* = 0.064) (Table 1(b)).

A trend towards a more active palliative therapy was seen with more patients being treated with chemotherapy over time among the three cohorts (*P* = 0.06). When comparing the impact on survival for those treated with chemotherapy, mainly gemcitabine (*n* = 45), the curves for Period A + B (*n* = 20) versus Period C (*n* = 25) were superimposed (*P* = 0.96) and median survival was six months.

A transcatheter arterial chemoembolization, or TACE, was administered during the last period to six patients. A median survival of 18 months for those patients was achieved.

A declining number of individuals got best supportive care (BSC). In Period A it was 69%, in B it was 66%, and in C it was 49% (*P* = 0.02). For these three cohorts, the survival curves were worse for Period C versus Period A + B (*P* = 0.04).

### 3.2. Perihilar Cholangiocarcinoma (PHC)

The diagnosis of PHC was histomorphologically established by brush cytology through an endoscopy or by biopsy in 80% of the patients. In the remaining 20% it was based on radiological findings.

Fifteen percent of patients with PHC were above 79 years of age and 8% were below 50 years.

No significant stage migration was identified over time in the 163 patients with PHC (Table 2(a)). Three patients were staged as T0 as their duct stricture was considered benign. These three patients were from the first period.

**Figure 3:**
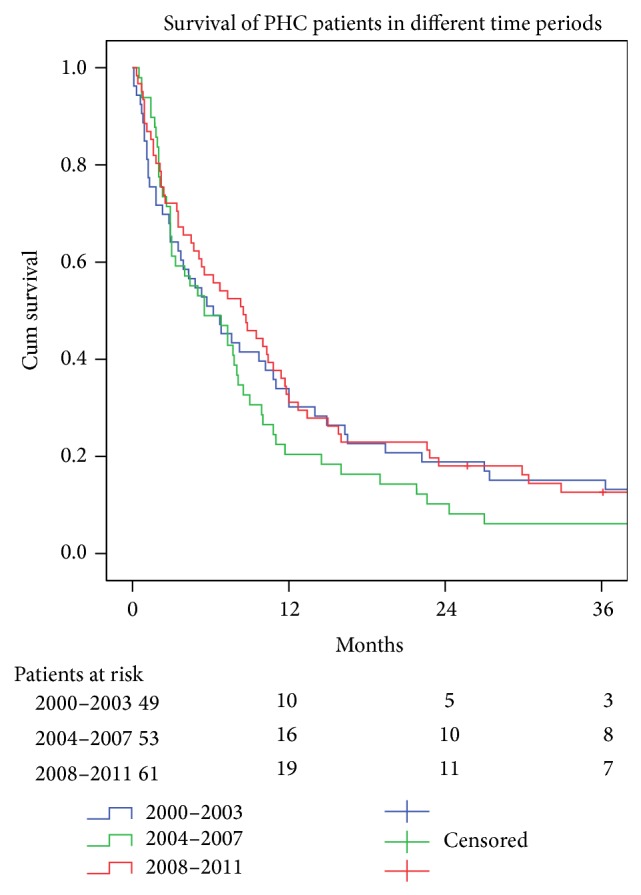
Survival curves (Kaplan-Meier log-rank test *P* > 0.38) for 163 patients with perihilar cancer (PHC) for three different time periods. Median survival (95% confidence interval) in months: 2000–2003, 5.50 (2.18–8.82); 2004–2007, 6.20 (2.84–9.56); 2008–2011, 8.50 (4.89–12.11).

The use of Bismuth criteria for staging the cancers in the confluence of the hepatic ducts increased over time from 49% in Period A to 85% in Period C (Table 2(a)). The median survival for the whole PHC-population was 6.8 months and 11% survived more than three years. There was no improvement in survival over time (Figure 3). Twenty patients had underlying liver disease—nine of them had PSC.

Fifteen percent of the patients had curatively aiming bile duct resection with a concomitant liver resection and 5.5% without liver resection. The three-year survival was 32% and 20%, respectively.

Six patients underwent liver transplantation. Three were transplanted according to the Mayo protocol, two of them had survived more than 3 years and three were transplanted outside the protocol and the longest survival was 23 months (Table 2(b)).

There was no increase in number of patients who received active palliative treatment (APT). In the first two periods there were 11 who were given miscellaneous palliative therapy (cox-2 inhibitor *n* = 9, sirolimus *n* = 2).

Median survival from date of diagnosis for the 34 patients that received APT was 8.8 months. The advanced stage of those with PHC is evident as approximately 50% were given best supportive care (BSC) in all periods.

## 4. Discussion

Biliary malignancies or cholangiocarcinomas are most often asymptomatic until late in the course of the disease and in an advanced stage when the diagnosis is established. In this analysis a great number of patients had their first doctor's consultation when their disease was at such an advanced state that only minor diagnostic procedures were motivated. Even in patients with advanced disease efforts to get a diagnosis were improved and, consequently, there was a decline over time in the number of individuals for whom diagnosis was established at a postmortem examination. During the study period CT and magnetic resonance imaging (MRI) improved differentiation between hepatocellular carcinoma and IHC [9]. For patients with PHC high-resolution magnetic resonance imaging (MRI) has enabled staging of a cancer in the bile duct confluence according to Bismuth's criteria and is the current preoperative standard to assess PHC with an accuracy around 50% [10].

MRI cholangiography is the golden standard used to delineate the localization and extent of a cancer and the possibility of curative surgery. It has a positive predictive value of 86% [11], but in a recent meta-analysis, Bismuth's criteria had a lower accuracy rate and were of no prognostic value in cases of PHC undergoing resection [9].

The decreasing number of individuals subjected to BSC over time mirrored the increased active therapeutic strategy for patients with IHC. The migration of patients to curative and active palliative therapy explains why survival for BSC patients in Period C was worse than in Period A + B.

The improvement in survival over time for patients with ICC could be explained by the fourfold increase in number of patients, from 3 to 12, who had curative surgery (liver resection and liver transplantation) from Period A to Period C contributing in total to 8 patients surviving more than 3 years and 6 to more than five years. If these patients subjected to curative surgery are excluded, there is no significant impact on the survival curves over time (*P* = 0.10). The three-year survival rate after curative surgery was thus 45%, a figure in agreement with what appears in recent reports [12].

The number of patients with ICC that underwent surgery was lower than expected. An explanation could be that in this study we study the whole cohort of patients not only those referred to the surgical clinic. More patients underwent curative surgery over time but the outcome for IHC patients who had curative surgery did not improve between Periods A and B (*n* = 10) versus Period C (*n* = 12). The improvement in survival in the last time period was only observed in those surviving longer than one year.

IHC was considered to be a contraindication for liver transplantation during the whole study period. In the 5 cases where liver transplantation was performed diagnosis was established first when the liver was explanted in two cases. A multi-institutional analysis in the Nordic countries reported a 5-year survival rate of 58% among those with IHC staged T1N0 and a CA 19-9 of less than 100 who underwent liver transplantation [13]. Based on this figure it has to be asked if there are more patients with IHC who could benefit from liver transplantation. As a recent meta-analysis shows that elevated CA 19-9-levels are associated with a worse prognosis independent of treatment [14], elevated CA 19-9-levels will remain a contraindication to transplantation; even the criteria were expanded.

In this material, preoperatively discovered lymph node metastases beyond the hepatoduodenal ligament were a contraindication for surgery. Even if lymph node metastases are a strong predictor of survival [15], the survival of IHC patients with lymph node involvement can be prolonged with hepatectomy and these cases should not be prematurely deemed noncurative [16].

There was a fourfold increase in number of patients with IHC who were treated with chemotherapy, mainly with gemcitabine. The survival outcome of this therapy did not change over time in this study; this is consistent with others' findings [17, 18].

Among patients selected for TACE in the last period of this study the median survival was 18 months. In comparison, median survival was six months for those receiving systemic chemotherapy. This difference could be explained by a better clinical stage in patients selected for TACE.

For those with PHC there was no improvement in the survival over the three time periods studied (Figure 3). There was no increase in the number treated with curative surgery (27% in Period A to 30% in Period C) and there was no increase in the number of individuals given active palliative treatment between the first and last periods. Despite progress with different treatment options, that is, liver transplantation [6] and chemotherapy [8], the number of patients who benefitted from these treatments was so small that it did not affect the survival rate of the entire population. Based on the present findings it seems reasonable to speculate that progress in treatment of PHC by surgical methods alone is limited. The Mayo protocol with irradiation and chemotherapy and consequent transplantation is reporting a five-year survival of 76% for individuals with PHC [7]. This is an example of how a combination of treatments can improve results. It also raises the question as to whether downsizing PHC and also IHC can increase the number of patients who can benefit from curatively aiming surgery. The role of adjuvant chemotherapy is still debated; a recent meta-analysis did not show any benefit [19], while a registry analysis identified that adjuvant chemotherapy was associated with significant survival benefits among patients with positive nodes or positive margins [20].

A more liberal use of chemotherapy during the last four-year period did not transfer into better outcomes. The presently used drug combinations that are considered effective [8] were not fully adopted even in the last time period; so there may be survival benefit still to be achieved. New protocols with innovative neoadjuvant and adjuvant therapies are needed to further improve long-term outcome following surgery or transplantation for patient with cholangiocarcinoma.

In conclusion, this analysis from a well-defined population in Sweden who had IHC or PHC has found an improvement in survival for patients with IHC. More individuals with IHC were over time treated with curative surgery or chemotherapy. Despite improvements in different treatment options, no changes in outcome were registered for patients with PHC during the first 12 years of this century.

## Figures and Tables

**(a):** 

Staging/year	2000–2003	2004–2007	2008–2011	Total
T0	1	0	0	1
T1	2	9	12	23
T2	2	0	4	6
T3	13	10	15	38
T4	1	1	3	5
N1	5	14	15	34
M+	17	19	34	70
Not staged	24	27	5	56

Total	65	80	88	233

**(b):** 

Treatment/year	2000–2003	2004–2007	2008–2011	Total
Curative aim				
Liver resection	2	6	9	17
Transplantation	1	1	3	5
Active palliative				
TACE	0	0	7	7
Chemotherapy	6	14	25	45
Miscellaneous	10	6	1	17
BSC	46	53	43	141

Total	65	80	88	233

**(a):** 

Staging/year	2000–2003	2004–2007	2008–2011	Total
T0	3	0	0	3
T1	2	5	2	9
T2	3	5	9	17
T3	1	3	5	9
T4	1	1	3	5
N1	9	11	10	30
M+	17	16	20	53
Not staged	13	12	12	37

Total	49	53	61	163

Bismuth staging				
1	13	8	11	32
2	1	0	6	7
3	5	6	14	25
4	5	5	21	31
Not staged	25	34	9	68

**(b):** 

Treatment/year	2000–2003	2004–2007	2008–2011	Total
Curative aim				
Liver resection + bile duct resection	5	11	9	25
Bile duct resection	6	1	3	10
Transplantation	2	0	4	6
Active palliative				
Chemotherapy	2	9	12	23
Miscellaneous	8	3	0	11
BSC	26	29	33	82

Total	49	53	61	163
